# Seasonal Variation in the Biochemical Composition and Fatty Acid Profiles of the Red Alga *Halymenia durvillei* from Ngazidja (Comoros)

**DOI:** 10.3390/molecules30061232

**Published:** 2025-03-10

**Authors:** Ahmed Radjabou Djoundi, Michèle Morançais, Aurélie Mossion, Emilie Ragueneau, Vony Rabesaotra, Helga Rim Farasoa, Vestalys Voahangy Ramanandraibe, Justine Dumay

**Affiliations:** 1 Institut des Substances et Organismes de la Mer, Nantes Université, ISOMer, UR 2160, F-44000 Nantes, France; djoundiahmed25@gmail.com (A.R.D.); michele.morancais@univ-nantes.fr (M.M.); aurelie.mossion@univ-nantes.fr (A.M.); emilie.ragueneau@univ-nantes.fr (E.R.); vony.rabesaotra@univ-nantes.fr (V.R.); 2 Laboratoire de Chimie et Valorisation des Produits Naturelles (LCVPN), Université d’Antananarivo, 101 Antananarivo, Antananarivo P.O. Box 906, Madagascar; rim.farasoa.helga@gmail.com (H.R.F.); voahangy.ramanandraibe@gmail.com (V.V.R.)

**Keywords:** *Halymenia durvillei*, biochemical, fatty acids, pigments, seasonal variations, nutritional indexes

## Abstract

The study of Comorian red alga *Halymenia durvillei* showed a significant biochemical composition with high ash and polysaccharide content and the presence of n-3 and n-6 essential fatty acid molecules. Seasonal monitoring showed a real change in biochemical composition depending on the harvesting period. On an annual average basis, the algae contained 35.59 ± 2.55% dw ashes, 0.7 ± 0.19% dw soluble proteins, 0.27 ± 0.02% dw total lipids, and 35.09 ± 6.14% dw polysaccharides. The pigment composition was 130 µg/g dw R-phycoerythrin, 1.49 µg/g dw chlorophyll a, and 0.09 µg/g dw carotenoids. The most abundant fatty acid identified was palmitic acid (C16:0), which accounted for almost 43.33% of total fatty acids. Oleic acid (C18:1n-9) was the most abundant unsaturated fatty acid, at 11.58%. Linoleic acid (C18:2n-6) was reported to be the most abundant polyunsaturated fatty acid in *Halymenia durvillei*. The fatty acid profile was also characterized by arachidonic acid (C20:4n-6) and eicosapentaenoic acid (C20:5n-3).

## 1. Introduction

Algae are globally distributed and colonize many marine aquatic habitats from temperate to tropical zones [1]. At the base of the trophic chain, they help to structure aquatic ecosystems [1,2]. They are found in different ecosystems such as estuaries, lagoons, salt marshes, and coral reefs, on rocky, muddy, sandy, or artificial substrates [1]. Thanks to their great biological diversity, marine algae are a source of interesting new active ingredients for the agro-food, pharmaceutical, and cosmetic sectors [3,4,5,6]. More than 15,000 original compounds have been chemically identified [7,8,9]. Today, macroalgae are mainly exploited through the production of phycocolloids [4,10], which are parietal polysaccharides in red (agars and carrageenans) and brown algae (alginates) [10,11]. They are used as texturizers in the agri-food, cosmetics, and medical sectors and to protect terrestrial plants against harmful organisms and the environment [12]. The study and use of seaweeds is becoming increasingly important worldwide [13,14,15]. Seaweeds are used extensively throughout the world, with 80% of harvests destined for direct or indirect human consumption. They contain large amounts of dietary fiber, proteins, and polysaccharides, as well as a wide variety of pigments and lipids [16,17,18,19]. The biochemical composition can vary according to species, geographical location, season, and temperature [11,17,20]. Seaweed is a good source of minerals, trace elements, and vitamins [16]. The protein content of algae is higher, particularly in red algae, and there are many different reserve polysaccharides depending on the species [16,19,21,22]. Seaweed has very low lipid concentrations, especially red seaweed. Although their lipid content is relatively low, algae play a key role in various fields, including biotechnology and nutrition. Indeed, the presence of essential polyunsaturated fatty acids in certain species is recognized for its beneficial effects on human health [16,17,23,24].

The Comoros have a predominantly aquatic surface with a rich and diverse algal flora [25,26]. The numerous upwellings of deep water allow the establishment of very diverse populations with significant local variation [25]. Little is known about the algae of the Comoros and very few species have been recorded to date. An in-depth study has enabled us to list a total of 81 taxa of algae described in Mayotte or more generally in the Comorian archipelago [27]. Of these, 15 belong to the brown algae phylum, 18 to the green algae phylum, and 48 to the red algae phylum. One of these algae species, *Halymenia durvillei* has interesting properties for human health and well-being [28,29,30,31].

*Halymenia durvillei* belongs to the Rhodophyta division, which is characterized by its intense red, non-gelatinous color, toothed texture, and repeatedly branched blades [32]. This alga is mainly distributed in the Indian-Pacific Ocean. Recent studies have examined its biochemical composition, highlighting the richness in polysaccharides, proteins, carbohydrates, minerals, fatty acids, and amino acids [19,33]. This type of red algae is a major source of carrageenan-type polysaccharides, used in the food and pharmaceutical industries [22,33]. It has also been shown to contain essential fatty acids considered beneficial to human health [19]. These properties make it a valuable resource for a healthy diet, helping to regulate metabolism and promote digestive health. The bioactive compounds produced, such as antioxidants, antivirals, and anti-cancer and anti-diabetic agents, are being studied for their potential in terms of human health [19,28,29,30,34,35,36]. *Halymenia durvillei* is also an excellent source of pigments for use in the food industry and human health [19,37].

The aim of this work is to study the biochemical composition of *Halymenia durvillei* in order to propose ways of exploiting this species of algae found on the Comorian coast. This is the first in-depth study of the chemical composition of a species of algae in the Comoros. The study of seasonal variation in the biochemical composition will provide a better understanding of the nutritional and economic potential of this alga, opening the way to solutions for the sustainable use of this resource on a local and international scale.

## 2. Results

### 2.1. Average Annual Biochemical Composition

The average annual biochemical composition of the red alga *Halymenia durvillei* is shown in Table 1. Ashes were the most abundant fraction, at 35.59 ± 2.55% of dry weight (dw). Total proteins constituted 13.93 ± 0.09% dw, and 0.74 ± 0.20% were water-soluble proteins. The water-soluble carbohydrate content was 1.52 ± 0.69% dw. Polysaccharides were more abundant, at 35.09 ± 6.14% dw. Lipid content was low, at 0.27 ± 0.02% dw. Chlorophyll a and carotenoids were minor pigments with levels of 1.49 ± 1.29 and 0.09 ± 0.07 µg/g dw, respectively. R-phycoerythrin was the major pigment, at 139.59 ± 103 µg/g dw.

### 2.2. Fatty Acid (FA) Analysis

The fatty acid composition is shown in Table 2. Saturated fatty acids (SFAs) were the primary fatty acids, at 55.1 ± 5.2% of the total fatty acids (TFAs). Unsaturated fatty acids consisted of 23.4 ± 2.4% monounsaturated fatty acids and 5.8 ± 3.0% polyunsaturated fatty acids. Unidentified fatty acids represented 15.7 ± 3.8% of TFAs.

Saturated fatty acids (SFAs) represented a variety of structures, with 11 different SFAs identified ranging from 0.1 to 43.33% of TFAs. Palmitic acid (C16:0) was the most abundant of the fatty acids. The monounsaturated fatty acid (MUFA) diversity consisted of six distinct MUFAs, dominated by oleic acid (C18:1n-9), at 11.58% of TFAs, followed by palmitoleic acid (C16:1), at 9.05% of TFAs. The main polyunsaturated fatty acids detected were linoleic acid (C18:2n-6), eicosapentaenoic acid (C20:5n-3), arachidonic acid (C20:4n-6), and ecosatrienoic acid (C20:3). The content of polyunsaturated fatty acids varied between 0.1% of TFAs for ecosatrienoic acid (C20:3) and 2.39% of TFAs for linoleic acid (C18:2n-6).

The nutritional quality indexes calculated with the fatty acids of *Halymenia durvillei* are presented in Table 3. The n-6/n-3 ratio was 2.84 and the C18/C20 PUFA ratio was low, at 0.69. The PUFA/SFA ratio recorded was 0.1. The atherogenic index (AI) and thrombogenic index (TI) were high, at 1.76 and 2.74, respectively. The hypocholesterolemic/hypercholesterolemic (h/H) ratio was low, at 0.38, the health promotion index (HPI) was 0.57, and the degree of unsaturation (UI) was 43.32.

### 2.3. Seasonal Variations

#### 2.3.1. Seasonal Climatic Conditions

**Figure 1:**
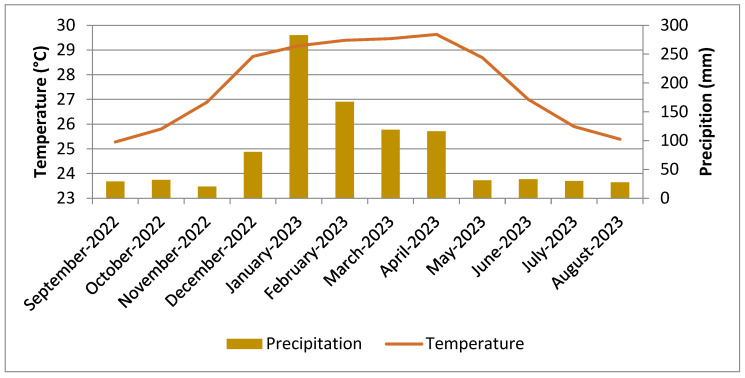
Average values for seawater precipitation and temperature at the harvesting site in Ngazidja (Mitsamiouli region).

The region where *Halymenia durvillei* was collected showed a difference in temperature and precipitation during the months of study (Figure 1).

During the summer and autumn in the Southern Hemisphere, precipitation and temperature were higher: precipitation varied from 282.7 mm in January to 116.25 mm in April, while temperature was around 29 °C, revealing a wet and hot climate.

During the winter and spring months, precipitation was low, at around 28.6 mm, and the temperature was around 26 °C (dry climate)

#### 2.3.2. Seasonal Biochemical Composition

**Figure 2:**
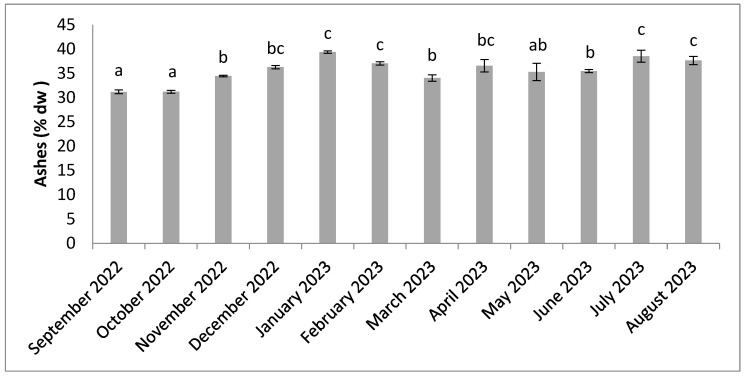
Seasonal variation in ash content. Significant differences are indicated by different letters (*p* < 0.05; n = 3).

**Figure 3:**
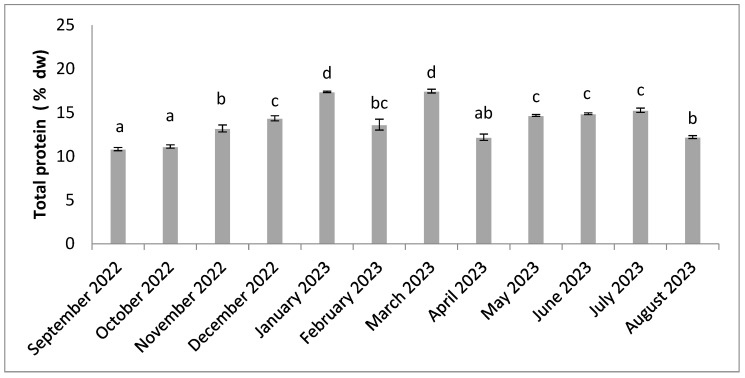
Seasonal variation in total protein content. Significant differences are indicated by different letters (*p* < 0.05; n = 3).

**Figure 4:**
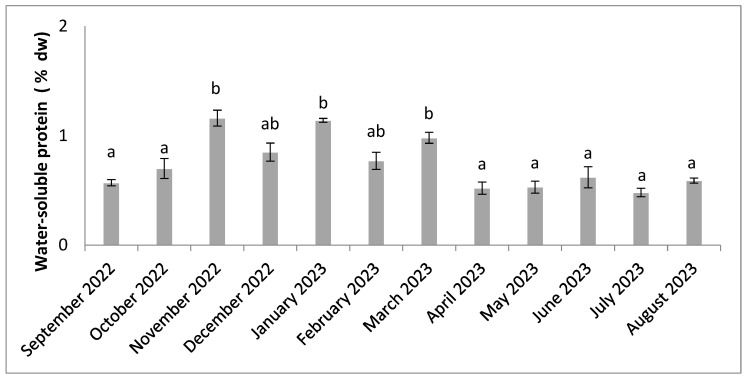
Seasonal variation in water-soluble protein content. Significant differences are indicated by different letters (*p* < 0.05; n = 3).

**Figure 5:**
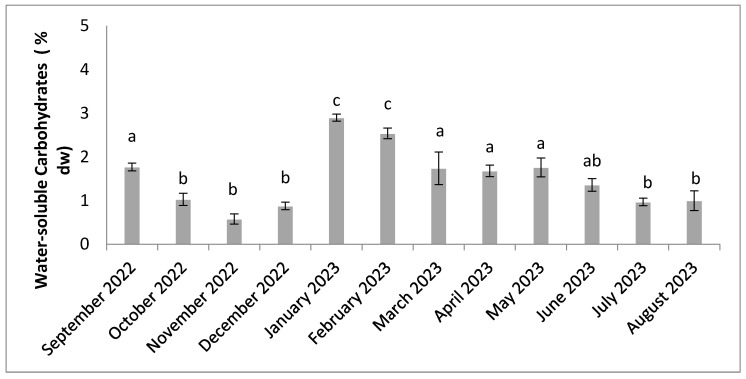
Seasonal variation in water-soluble carbohydrates content. Significant differences are indicated by different letters (*p* < 0.05; n = 3).

**Figure 6:**
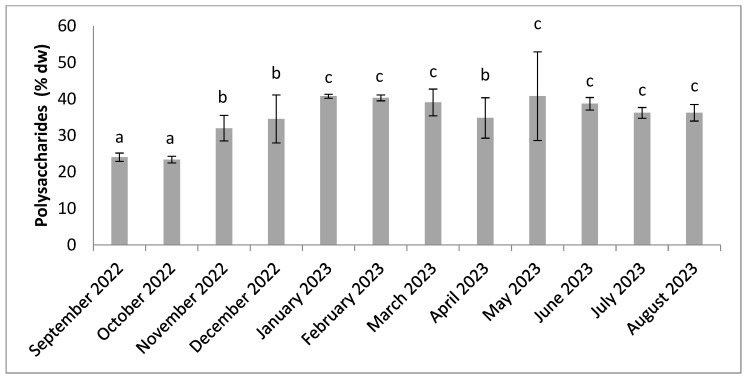
Seasonal variation in polysaccharides content. Significant differences are indicated by different letters (*p* < 0.05; n = 3).

**Figure 7:**
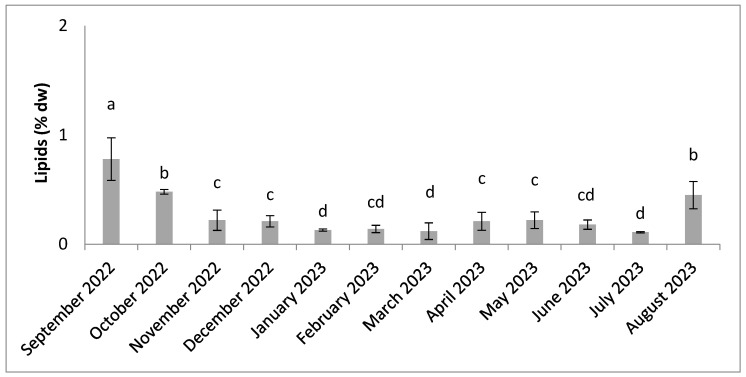
Seasonal variation in lipids content. Significant differences are indicated by different letters (*p* < 0.05; n = 3).

**Figure 8:**
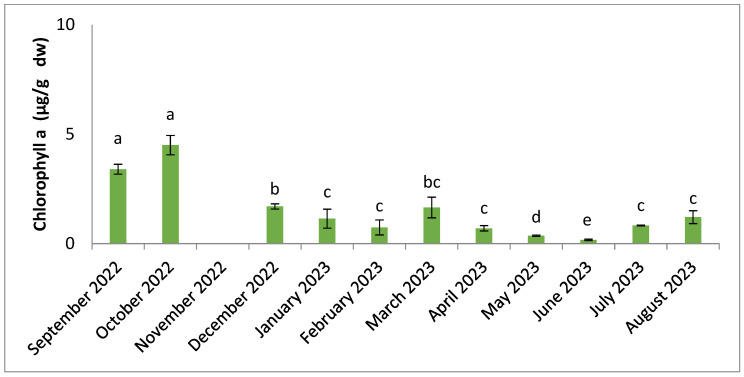
Seasonal variation in chlorophyll a content. Significant differences are indicated by different letters (*p* < 0.05; n = 3).

**Figure 9:**
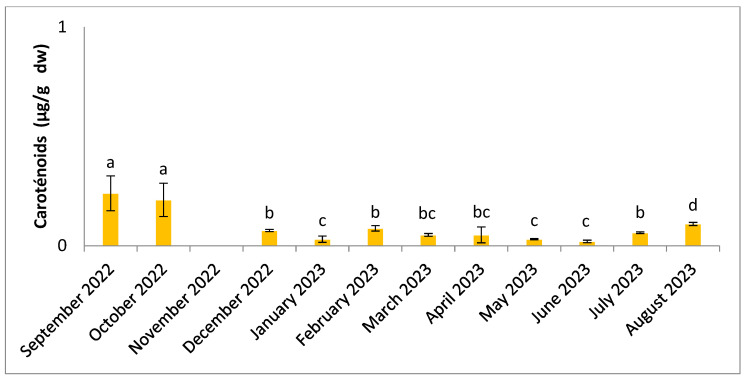
Seasonal variation in carotenoids content. Significant differences are indicated by different letters (*p* < 0.05; n = 3).

**Figure 10:**
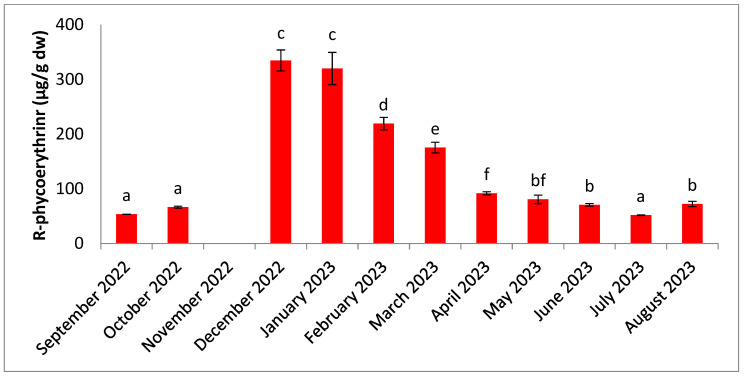
Seasonal variation in R-phycoerythrin content. Significant differences are indicated by different letters (*p* < 0.05; n = 3).

Figure 2, Figure 3, Figure 4, Figure 5, Figure 6, Figure 7, Figure 8, Figure 9 and Figure 10 show the annual variation in the biochemical composition of *Halymenia durvillei* in terms of ashes, proteins, carbohydrates, polysaccharides, and lipids. The ash content (Figure 2) varied between 31.18% and 38.52% dw. The ash contents recorded show significant differences (*p* < 0.05) according to the results of the statistical analysis (Kruskal–Wallis and Dunn’s post hoc test). The lowest ash levels were recorded between September and October. From November, ash levels are higher until August, with little change.

Total protein contents ranged from 10.83% in September and 17.48% in March (Figure 3). The lowest total protein levels were observed in spring, September and October, and the highest levels were in January and March. There were statistical differences (n = 3, *p* < 0.05) throughout the twelve studied months.

Water-soluble protein levels were generally low, with a maximum in January (1.16% dw) and a minimum in April (0.48% dw) (Figure 4). Water-soluble protein levels were low between September and October and April and August, while they were higher between November and March.

Water-soluble carbohydrate levels varied between 0.58% dw and 2.91% dw (Figure 5). The highest levels were recorded in January and February, and low levels between October and December. The results of the statistical analysis showed significant differences (n = 3, *p* < 0.05) between the months.

The polysaccharide contents showed a seasonal variability of 23.39% to 40.77% dw during the studied period (Figure 6). A significant difference (n = 3, *p* < 0.05) was observed, with a lower content in September and October and a higher content in November.

The results (Figure 7) showed low levels of lipids, ranging from 0.13% dw to 0.83% dw. The seasonal variation was observed, with statistically significant differences (n = 3, *p* < 0.05) between months. The highest lipid content was recorded in September. The lowest lipid values were recorded between January and March and in July.

Figure 8 and Figure 9 shows the seasonal changes in photosynthetic pigment content. The results of the statistical analysis (ANOVA, Tukey) showed significant differences (*p* < 0.05) between months for carotenoids and chlorophylls. The higher values were obtained in the dry season, September for carotenoids and in October for chlorophyll a (0.24 µg/g dw and 4.50 µg/g dw, respectively), and lower values were found in the hot season in June (0.02 µg/g dw for carotenoids and 0.17 µg/g dw for chlorophyll a).

The variation in R-phycoerythrin concentration is shown in Figure 10. It ranged from 51.72 µg/g dw in July to 334.60 µg/g dw in December. The results of the statistical analysis showed significant differences (n = 3, *p* < 0.05) between the months. Concentrations were highest during the dry season and lowest during the hot season.

#### 2.3.3. Variations of Fatty Acid Composition

The variation in fatty acid composition is shown in Table 4. Saturated fatty acids ranged from 47.46% to 63.37% of total fatty acids and were the most abundant group. Monounsaturated fatty acids ranged from 20.43% to 27.26%, and polyunsaturated fatty acids from 0.6 to 11.88%, of total fatty acids. Saturated fatty acids were higher from May to December and lowest from January to April. Monounsaturated fatty acid levels were highest from February to April and lowest in December and from May to August. Polyunsaturated fatty acid levels were highest in December and lowest in August.

Palmitic acid (C16:0) was the main saturated fatty acid throughout the harvest, with levels ranging from 29.97% of total fatty acids in January to 56.63% in August, followed by stearic acid (C18:0). Monounsaturated fatty acids were dominated by oleic acid (C18:1n-9), followed by palmitoleic acid (C16:1n-7), with levels varying according to the harvested period. Oleic acid (C18:1n-9) was the main monounsaturated fatty acid throughout the year, except in March and April, when palmitoleic acid (C16:1) was dominant. The profile of polyunsaturated fatty acids varied depending on the harvest period. Linoleic acid (C18:2n-6) was more abundant from January to June, while arachidonic acid (C20:4n-6) and eicosapentaenoic acid (C20:5n-3) were more abundant from September to December. A predominance of n-6 polyunsaturated fatty acids over n-3 polyunsaturated fatty acids was observed throughout the year.

The n-6/n-3 ratios ranged from 1.3 to 14.22 (Table 5), with low values from October to December. January was the only month in which the n-6/n-3 ratio was higher, at over 10. The C18/C20 PUFA ratios ranged from 0.6 to 3.14 in August and January, respectively. The values recorded for PUFA/SFA ratios were very low, ranging from 0.01 in August to 0.21 in January. AI indices ranged from 1.25 to 2.85, with the lowest values recorded from December to April. TI values were much higher, ranging from 1.75 to 5.06. The h/H ratios ranged from 0.17 to 0.55, with the highest values recorded between September and January. HPI indices ranged from 0.35 to 0.81, and UI rose from 23.03 to 70.71.

#### 2.3.4. Principal Component Analysis of Fatty Acids

**Figure 11:**
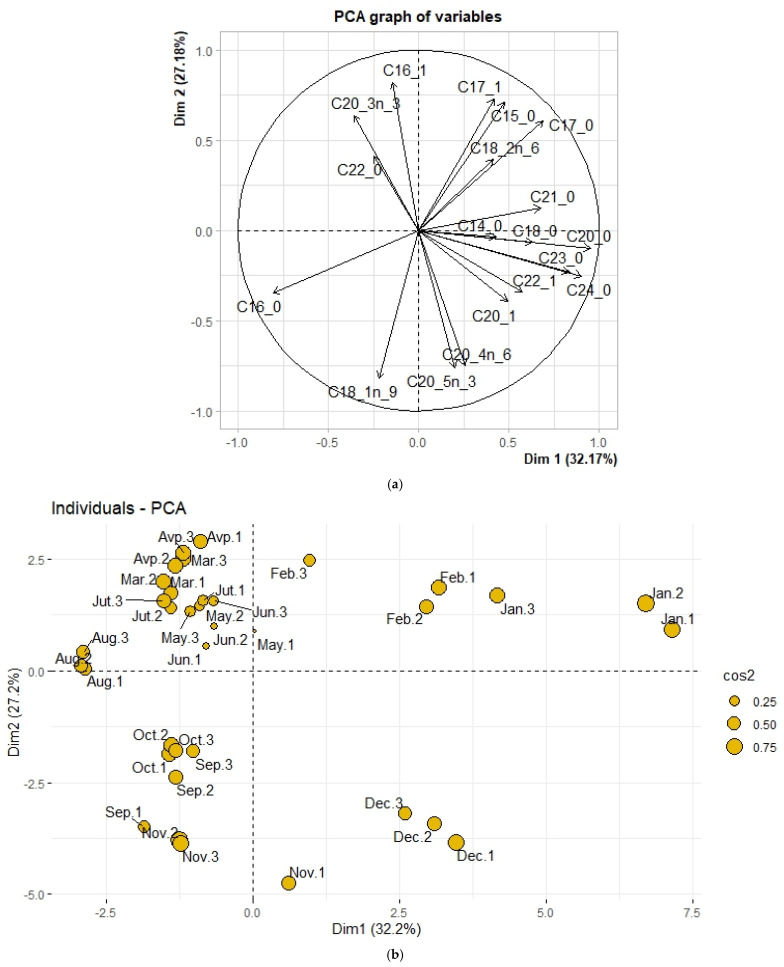
Correlation circle (**a**) and scatter plot (**b**) representing fatty acid composition scores from principal component analysis.

Principal component analysis (PCA) was performed to gain insight into the similarities and differences in the fatty acid profiles of the samples collected (Figure 11). The analysis was performed on the fatty acid composition data in triplicate for each month. Fatty acids with small variations were removed from the principal component analysis. The variance contribution of the first two dimensions exceeded 50%, with 32.17% for dimension 1 and 27.18% for dimension 2.

The results of the principal component analysis show that the samples are distributed according to the harvest period. A group of samples from September to December is distributed in the two lower quadrants. Although all samples from September to August are distributed in the upper two quadrants, two groups seem to be formed here. The January and February samples are grouped in the upper left quadrant, while the April to August samples are in the opposite right quadrant.

In the correlation circle, palmitic acid (C16:0) is projected on axis 2 and associated with the August samples. On the other hand, oleic acid (C18:1n-9) is projected on axis 1 and is well associated with the September, October, and November samples. Arachidonic acid (C20:4n-6) and eicosapentaenoic acid (C20:5n-3), projected on axis 1 at a much-reduced angle, are associated with the December samples. Eicosenoic acid (C20:1) and erucic acid (C22:1) also seem to be associated with December samples. Behenic acid (C22:0), eicosenoic acid (C20:3), and palmitoleic acid (C16:1), projected on axis 2, are well associated with samples from April to July. In the upper left quadrant, linoleic acid (C18:2n-6), heptadecanoic acid (C17:0), and pentadecanoic acid (C15:0) are associated with February samples. In addition, lignoceric acid (C24:0), tricosanoic acid (C23:0), heneicosanoic acid (C21:0), arachidic acid (C20:0), and stearic acid (C18:0), projected along axis 1, are associated with the January samples.

## 3. Discussion

### 3.1. Average Annual Biochemical Composition

The ash content of *Halymenia durvillei* in the present study is in agreement with previous studies on the same species (39.51–41%) [19,33]. However, the ash content is lower than that reported for *Halymenia floresii* (50.2%) and higher than that obtained for *Halymenia dilatata* (21.92%), *Halymenia maculata* (23.61%), and *Halymenia porphyriformis* (17%*)* [24,33,38].

The total protein content recorded is similar to that of another study on *Halymenia durvillei* from the Philippines (13.02%) [33]. It is also comparable to that reported for *Halymenia maculata* (11.1%), *Halymenia dilatata* (9.9%), and *Halymenia florisei* (9.4%) [24,33]. For water-soluble protein content, Magdugo et al. reported amounts of 5.3% and 5.5% for *Halymenia durvillei* and *Halymenia dilatata*, respectively, which is higher than that found in the present study [19].

Concerning water-soluble carbohydrates, the average content in the present study is lower than that already reported in the literature for the same species [39]. It is also lower than that recorded for *Halymenia dilatata* [19]. A similar result in terms of soluble carbohydrate content was reported for *Grateloupia turuturu* [39]. On the other hand, in other species of red algae, the water-soluble carbohydrate content is very high, as in the case of *Solieria chordalis* [40].

The polysaccharide content is much higher than in previous studies of the same species (16–28.4%) [22,33]. It is also higher than that reported for *Halymenia maculata*, *Halymenia porphyroides, Halymenia floresii*, and *Halymenia dilatata* [24,25,37,41]. It is possible to extract more polysaccharides with pre-treatments, but this may alter the appearance and osidic composition [22].

The lipid content is very low compared to that reported in the literature for *Halymenia durvillei* (1.29–7.1%) [19,33]. It is also lower compared with other *Halymenia* species. Garcia et al. reported a value of 12.3% for *Halymenia floresii* [24] and Choudhary et al. reported a value of 1% for *Halymenia porphyriformis* [38]. However, Kho et al. obtained the same content of lipids for *Halymenia dilalata* [33].

The pigment contents of *Halymenia durveilli* are relatively low in our study compared to those obtained by Magdugo et al. on the same species [ ]. Higher concentrations of chlorophyll and carotenoids have also been reported in *Halymenia florisei* and *H. porphyroides* [41,42,43]. Carotenoids are well known for their ability to improve skin defense against UV rays [44]. Carotenoids can play important roles in human health, such as preventing eye disease, boosting immune defenses, and reducing the incidence of inflammatory and cardiovascular problems and diabetes [44,45,46]. As for chlorophylls, they are known as free radical scavengers and have antimutagenic effects with their bioactivities against obesity, inhibition of viral infections, and anti-cancer effects [44,47]. The R-phycoerythrin (R-PE) concentration of our extract is higher than that of a previous study of the same species [48]. For *Halymenia florisei*, a higher R-phycoerythrin concentration has been reported, i.e., a 20-fold higher R-phycoerythrin concentration [42,49]. Several other species of red algae, such as *Kappaphycus alvarezii*, *Grateloupia turuturu*, *Mastocarpus stellatus*, and *Gracilaria gracilis* recorded higher R-phycoerythrin concentrations than our results [17,50,51]. R-phycoerythrin is widely used as a natural coloring agent in the food industry and is known as an antioxidant molecule that can reduce the formation of cancer cells in the human body [52].

Although the lipid content of *Halymenia durvillei* is relatively low, analysis of its fatty acid profile remains relevant. This alga may contain polyunsaturated fatty acids (PUFAs), such as omega-3 and omega-6, which play a key role in human and animal nutrition [16]. In addition, red algae are known to contain long-chain fatty acids that are of interest for cardiovascular health and neuronal development [9,16,17,23]. The fatty acid profile also provides valuable information about the algae’s lipid metabolism and its adaptation to environmental conditions [17,53,54]. In terms of applications, certain essential fatty acids, even if present in small quantities, are of interest in sectors such as agri-food and pharmacology. Profiling the fatty acids in this species will provide information about its biochemical composition and potential applications.

The study conducted by Magudo et al. carried out on *Halymenia durvillei* shows comparable levels of saturated fatty acids (51.15%), lower levels of monounsaturated fatty acids (15.75%), and higher levels of polyunsaturated fatty acids (9.81%) than our results [19]. The composition of saturated fatty acids is higher than that recorded for *Halymenia dilatata* (31.9%), *Halymenia ceylanica* (37.56%), and *Halymenia porphyraeformis* (39.2%) [18,19,55]. Two studies reported values of 18.8% and 68.93% for *Halymenia floresii* [24,56]..

The monounsaturated fatty acid content is in line with that reported for *Halymenia dilatata* and *Halymenia ceylanica*, and lower than that obtained for *Halymenia floresii* [19,24]. As for the polyunsaturated fatty acids, the content is very low compared to that recorded in *Halymenia porphyraeformis* (52.7%) and *Halymenia sinensis* (42.2%), but comparable to that obtained for *Halymenia floresii* (6%) [18,56]

The palmitic acid (C16:0) content is similar in the present work to that reported for *Halymenia durvillei* in the literature [19]. In *Halymenia porphyroides*, however, the predominant fatty acid is stearic acid (C18:0), which is higher than that recorded in the present study [41]. Oleic acid (C18:1n-9) has a very high value compared to that reported in the study of the same species, in which myristoleic acid (C14:1) was found to be the main monounsaturated fatty acid [19]. However, the level of oleic acid content is comparable to that reported for *Halymenia dilatata* and lower than that reported for *Halymenia porphyroides,* and was not reported for *Halymenia florisei* [19,24,41]. The linoleic fatty acid molecule (C18:2n-6) has a higher value than in the study on the same species [19]. The linoleic acid composition (C18:2n-6) is also higher than in *Halymenia dilatata* and lower than in *Halymenia florisei* [19,24].

*Halymenia durvillei* also contains polyunsaturated fatty acids such as arachidonic acid (C20:4n-6) and eicosapentaenoic acid (C20:5n-3), which have not been reported in the literature for the same species [19]. Eicosapentaenoic acid was detected in *Halymenia florisei* with a value comparable to our result [24]. Arachidonic acid has been reported at low levels in *Halymenia venusta* [57]. These two essential polyunsaturated fatty acids (arachidonic and eicosapentaenoic) have been reported in red algae such as *Grateloupia turuturu*, *Pyropia dioica*, *Gracilaria gracilis*, and *Palmaria palmata* [16,17,23,58]. The fatty acid composition reveals the presence of essential polyunsaturated fatty acids known for their beneficial effects on health, in particular their effect on immune regulation, cancer prevention, and cardiovascular problems [16,17,58,59,60,61]. In addition, linoleic acid (C18:2n-6) and oleic acid (C18:1n-9) are considered highly nutritional due to their protective role against cardiovascular disease [53].

The annual n-6/n-3 ratio is slightly lower than that already reported in a previous study of *Halymenia durvillei* and confirms its benefits as a suppressant of some health problems [19,62]. In *Halymenia dilatata*, a lower n-6/n-3 ratio (0.46) has already been recorded for some species of red algae [17,19]. On the other hand, other red algae such as *Halymenia florisei* have reported very high n-6/n-3 ratios [16,24]. Unlike our study, *Halymenia dilatata* has a higher C18/C20 PUFA ratio. *Halymenia durvillei* has a PUFA/SFA ratio below the minimum value recommended by the Food and Agriculture Organization/World Health Organization (FAO/WHO). A PUFA/SFA ratio of less than 0.45 is undesirable as it increases blood cholesterol levels [62]. The AI, TI, and h/H indices are considered to be analytical indicators for assessing the nutritional quality of the fatty acid profile and the effects of fatty acids on human health [63]. The AI index measures the proportion of pro- and anti-atherogenic TFAs, and the TI index shows the tendency to form clots in blood vessels [60]. The annual value of the AI index is comparable to that of *Halymenia durvillei* and higher than that of *Halymenia dilatata* from the Philippines [19]. As for the TI index, its value is higher than that reported in *Halymenia durvillei* and *Halymenia dilatata* from the Philippines [19]. A high h/H ratio is considered more beneficial to health [19,63]. Our result shows a low h/H ratio, like that observed in *Halymenia durvillei* by Magdugo et al. [19], but it can be considered important from a nutritional point of view [63]. The health promotion index (HPI) is in the range of 0.16 to 0.68, as reported in the literature, mainly for dairy products such as milk and cheese [64].

The annual biochemical composition provides an overall estimate of the body’s biochemical characteristics. However, it only reflects overall trends and does not consider the occasional fluctuations that can occur at different times of the year. The biochemical composition for the year as a whole therefore takes seasonal fluctuations into account, allowing us to understand variations linked to environmental factors. The biomass used for seasonal monitoring in this study was collected between September 2022 and August 2023. Seasonal monitoring makes it possible to identify dips in biochemical composition, which may be essential for ensuring sustainable use.

### 3.2. Seasonal Monitoring

The seasonal biochemical composition of seaweed shows variability that can be related to several factors, in particular, geographical location, the origin of the species, growth, and environmental conditions [16,17,40,65]. Studies on the variability of biochemical composition are essential for identifying the most interesting seasons for using a given species of algae.

There were slight but significant differences in ash content between the months studied (Figure 2). The increase in ash content observed from November onwards could be related to a high concentration of minerals in the algae tissues in response to environmental conditions. In general, the ash content is very high and seems to exceed that reported in some studies on red algae, which give values between 10 and 30% [66]. The ash levels found are consistent with the variability of some algae, such as the species *Hypnea flagelliformis* [17,20,67]. These levels are within the range of values often recorded in red algae [8] and imply a considerable mineral content [67,68].

Total protein levels show greater variability, with higher levels during the warm, wet period from December to March (Figure 3). This result can be explained by the heavy rainfall, which washes large quantities of fresh water loaded with nitrogenous nutrients on to the land and into coastal waters. Proteins are synthesized from the nitrogen present in the environment [40]. The level of water-soluble proteins, which make up a specific fraction of total proteins, also tends to be higher in hot, humid periods (Figure 4). Higher protein levels have already been observed during rainy periods in *Solieria chordalis* [40]. A lot of studies on algae indicate that the protein content is lower in summer and higher in winter [69,70]. In addition, lower protein values may be related to the reduced availability of nitrogen compounds. Protein accumulation also depends on the season and origin of the algae [17,23,40]. Research has already shown that protein levels are higher when the algae are young and developing [71]. The total protein content is comparable with the values often reported for red algae [72]. They have multiple bioactivities, notably as anti-mutagens, blood-sugar-lowering agents, calcium precipitation inhibitors, cholesterol-lowering agents, antioxidants, and liver function enhancers [73]. However, protein composition depends on the season and environmental parameters, such as nutrient content. Nutrient availability depends on biotic and abiotic factors. It is therefore important to take into account factors such as temperature, light, desiccation, carbon availability, salinity, and water movement [20]. The levels obtained in our study are significant, but in seaweed, these levels can be as high as 47% by dry weight [69].

Water-soluble carbohydrate levels show a marked seasonal dynamic, with significant differences (Figure 5). Their accumulation is most pronounced in the middle of the hot, humid period (January–March), when the temperature rises considerably. Very high levels of water-soluble carbohydrates are also recorded with increasing temperature in a number of studies cited in the peer-reviewed literature [20,40]. The water-soluble carbohydrates content may increase as a result of high light intensity, indicating high-efficiency photosynthetic production [20,74]. In addition to the influence of environmental factors, high accumulation may be linked to the stage of growth [1]. Indeed, water-soluble carbohydrates content is higher during the stationary phase of growth, a phenomenon described by various marine algae [75]. Furthermore, sugar production can be influenced by the genetic distinction between algal species, which leads to variations in sugar production [39,40].

Polysaccharides also show a marked seasonal variation (Figure 6). Levels are highest between November and August, which could be explained by an intensification of photosynthesis and carbon metabolism. Polysaccharide levels vary throughout the year, with values higher than those found in several studies on red algae [40]. It is during periods of heavy rainfall that higher levels of polysaccharides are observed, which may be favored by the migration of nitrogenous particles into the seawater [70]. The variations observed in polysaccharide content can be linked to the influence of complex exogenous factors, as well as endogenous changes in the organisms. Polysaccharide levels change as a function of reproduction: the more mature the algae, the higher the polysaccharide content [11,40]. Between January and March, a high accumulation of polysaccharides is recorded, indicating the maturity phase [76]. In fact, the high polysaccharide values are obtained during the period of full algal growth [11]. However, polysaccharide levels start to decrease in April when the algae reach their maximum abundance [77]. Exogenous factors influence the growth rate of the algae, the morphology of the thallus and trigger reproduction and germination. Accumulating polysaccharides is part of a dynamic linked to the algae’s lifecycle. During the juvenile phase, a large amount of energy is allocated to the synthesis of structural and reserve polysaccharides. During this phase, polysaccharide production is slightly lower as the algae retain many growth nutrients [40]. From the vegetative phase onwards, some of the stored polysaccharides are mobilized to provide the energy needed to form spores and mature reproductive structures. Yields remain relatively constant during this phase and begin to increase as the algae reach maturity [11]. During the reproductive phase, some of the accumulated polysaccharides are mobilized to support the development of reproductive structures and spore formation. Reproduction requires a significant amount of energy, so polysaccharide reserves are depleted during this period. As a result, polysaccharide levels increase as the algae develop.

Higher polysaccharide contents are reported in *Kappaphycus alvarezii* in the high season with young thalli and in the low season with mature thalli [78]. In addition, it is reported in the literature that polysaccharide contents are higher in apical segments than in basal segments [79]. Growth-related physiological and structural functions may therefore control polysaccharide biosynthesis [79]. Research carried out on *Kappaphycus alvarezii* has shown that, apart from the harvesting season, distance from the coast has a considerable influence on the carrageenan-type polysaccharide content of seaweed [80]. The high polysaccharide content is also a very important factor in the algae’s resistance to a high salinity environment, maintaining the ionic balance in the cell [81]. High solar light intensity in the algal growth environment promotes the rate of photosynthesis and the increase in polysaccharide content [82]. Photosynthesis allows algal cells to absorb nutrients that are converted into polysaccharides, which are located in the cell wall [83]. According to several articles, the polysaccharides of *Halymenia durvillei* belong to carrageenans and form a compound of major interest. It represents a significant proportion of the dry weight of this seaweed compared with other compounds. This richness in carrageenan represents a very high economic value for *Halymenia durvillei*. Carrageenans are compounds that can be used for many applications, including the treatment of inflammatory diseases, wound healing, and action against enveloped *Herpesviridae* viruses, and with antitumor potential against colorectal cancer stem cells [84,85].

Regarding lipids, the results obtained confirm the range generally found in marine algae [86,87]. The lipid content shows significant variations between the months studied, with high values in September and October when temperatures are low (Figure 7). The lipid content decreases when the temperature rises significantly from November onwards, before increasing slightly in August when the temperature drops again. In fact, lipid levels are higher when cells are exposed to low temperatures [70,88,89,90,91].The higher lipid levels recorded in September indicate an optimal response to light and temperature that promotes enhanced thylakoid stacking [70]. The variation in lipid content is comparable to that observed in some studies of red algae [5,92]. These lipid levels are lower than in other annual studies of red algae [17,54,66,87]. Seasonal variability in lipid content is also associated with other factors, such as the reproductive phase [5,93]. The statistically significant differences between the months studied cannot be attributed solely to the factors, but can also be explained by the stage of the biological cycle [20]. Lipid content also varies according to the taxon, and its composition is not found most of the time under optimal growth conditions for biomass production [69]. The lipid content is very low in our results and can be considered advantageous for nutrition [20]. *Halymenia durvillei* has low lipid content and can be considered a low-fat food alga.

The results show a significant variation in the concentrations of chlorophyll a, carotenoids, and R-phycoerythrin over the course of the year.

Levels of chlorophyll a and carotenoids are highest during the period of lowest temperatures, in September and October (Figure 8 and Figure 9). The lowest concentrations are recorded when the temperature starts to rise from December onwards. Several studies have already highlighted the presence of low concentrations of photosynthetic pigments when temperatures are high, while they increase with lower temperatures [94,95]. According to previous research, algae show maximum development of the light absorption complex at low temperatures [94]. Concentration levels of these pigments are highly dependent on illumination, and adaptation to low light levels can result in high concentrations [51,95,96,97]. Light plays an important role in the synthesis of algal pigments, regulated by photoreceptors that absorb light at different wavelengths [97]. Thus, the variation in these pigments depends not only on temperature, but also on other environmental parameters, such as light intensity and nutrient availability. Chlorophylls and carotenoids are known for their DPPH (2,2-diphenyl-1-picrylhydrazyl) radical-reducing effects, as antioxidants and in food processing [98]. Among the carotenoids, there is fucoxanthin, which may have antitumor, anti-diabetic, anti-inflammatory, hepatoprotective, anti-angiogenic, anti-malarial effects and prevent cardiovascular problems [99]. Chlorophylls in algae have other ranges of biological activity, including anti-cancer activity, and could potentially serve as a source of magnesium [44]. In our study, the variation in these pigments shows low levels, which may be influenced by the pre-treatment and extraction methods used. The preservation of these compounds requires the demonstration of other protocols, such as pre-treatment using freeze-drying.

R-phycoerythrin concentrations are low in September and October, then increase considerably during the period of intense rainfall (Figure 10). From April onwards, concentrations fall, which could be due to the consumption of reserve substances during the period of heavy rainfall, as well as to the release of tetraspores [94]. Like all phycobiliproteins, R-phycoerythrin is potentially consumed as a source of nitrogen, and its reduction can be explained by high light exposure [74]. Concentrations of R-phycoerythrin may also depend on sample pre-treatment and extraction procedures [44]. Samples are thoroughly air-dried in the dark before being cryo-ground and extracted at low temperatures with a sodium phosphate buffer. This technique, which combines grinding and freeze–thawing, is considered suitable for higher yields [17,44]. Low R-phycoerythrin levels are already linked to the destruction of phycobiliproteins related to environmental physicochemical parameters and origin [51,95,96,100]. pH is considered to be one of the factors affecting the destruction of phycobiliproteins, and it is recommended to handle pigments at their optimal pH [101,102,103]. R-phycoerythrin has previously shown antioxidant activity by scavenging peroxyl radicals in vitro [104]. This pigment has also already demonstrated significant antioxidant activity in ABTS (2,2′-azino-bis(3-ethylbenzothiazoline-6-sulphonic acid) and FRAP (reduced iron antioxidant power) tests, while showing significant cytotoxicity against the human hepatocellular carcinoma cell line [52]. Thanks to its antioxidant properties, R-phycoerythrin can be used to prolong food preservation. It can be included in food supplements for its health benefits.

The variability in fatty acid composition shows a richness in saturated fatty acids, followed by monounsaturated and then polyunsaturated fatty acids (Table 3), as in most seasonal studies of red algae [17,105,106,107]. Contrary to this study, the increase in polyunsaturated and monounsaturated fatty acids with a decrease in saturated fatty acids has already been highlighted in the literature [20]. Nevertheless, the percentages of polyunsaturated fatty acids are very low compared with those recorded in certain seasonal studies on red algae [17]. Seasonal temperature changes have a major effect on the fatty acid composition of cell membranes [108]. The content of saturated fatty acids is higher than that of unsaturated fatty acids due to the higher water temperatures [70]. Water temperature is one of the main factors that can influence the content of fatty acids, especially unsaturated fatty acids [54,109]. The most dominant fatty acids throughout the seasons are palmitic acid and oleic acid, as found in a previous study on *G. turuturu* [17]. However, the dominance of fatty acids other than palmitic acid (C16:0) has been reported in some seasonal monitoring studies [20,110]. Arachidonic (C20:4n-6) and eicosapentaenoic (C20:5n-3) acids are reported throughout the harvesting period but at lower levels than in some red algae [17,105,111,112,113]. The higher levels of these two fatty acid molecules between September and December could be linked to the low temperatures recorded during this period and other biological factors. This variation in fatty acid molecules may be related to species origin, stress conditions, and life cycle stage [88,89,90,91,114].

The n-6 polyunsaturated fatty acid content is well represented during the warm season (May–June), as shown by a study on the *G. turuturu* species [17]. Studies on red algae show a predominance of n-3 polyunsaturated fatty acids compared to n-6 polyunsaturated fatty acid types, contrary to our results [17,112]. The n-6/n-3 ratios obtained in our study are in line with international standards, with the exception of the ratio recorded in February [62]. Ratios of n-6/n-3 below 10 may have suppressive effects against several diseases [115,116]. These ratios of n-6/n-3 fatty acids > 1 may vary depending on the species, environmental conditions, and season [24]. The low ratios recorded between September and December are the most important for preventing diseases such as cardiovascular problems and other illnesses [115,116].

Between September and December, the C18/C20 PUFA ratios are much more favorable than those during the rest of the year. The PUFA/SFA ratios are very low throughout the year, yet it is with a high ratio that the effect is positive [64]. Following this reasoning, it is therefore the samples from October to January that have higher positive effects than the rest of the harvest. The highest AI indices are recorded between January and April and are comparable to the values reported for fish, meat, and milk [64]. The IT indices recorded between October and April are within the range of values reported in the literature for most red algae [18,64]. On the other hand, the very high IT indices of our result are also reported in some bibliographic information [18]. A fatty acid composition with lower IA and IT indices has a better nutritional quality, but no organization has yet provided official recommended values [18,64]. The significant h/H indices are higher and were recorded between September and December. All the h/H indices obtained are very low compared with the seaweed literature [64]. On the other hand, results on seafood and dairy products reported indices comparable to those of our study. High HPI values are thought to be better for human health. In this sense, samples from December to April are the most beneficial [64]. Nevertheless, this HPI has a shortcoming that requires reliable evidence to optimize the relevant coefficients. The UI values of our samples are comparable to some previous results [18,64]. Information suggests that a high degree of instauration can maintain fluidity at a relatively low temperature [64].

The fatty acid composition of *Halymenia durvillei* clearly shows seasonal variability, as shown by other studies [17,18,54,113]. PCA analysis (Figure 11) shows that groups of samples with similar fatty acid profiles cluster together according to season [107]. However, small deviations were observed within each group, probably due to the sampling and pre-treatments used. In addition, this study shows a very clear distribution between the samples, which allows a better description of the different seasons. These results show that each sample can be distinguished based on its fatty acid composition. In fact, we can see that the samples from September to November in 2022 are grouped in the two lower quadrants, unlike those from 2023. This allows us to confirm that the fatty acid profile can vary from one year to the next. As a result, fatty acid levels appear to be higher in the dry season than in the warm wet season. This result is not consistent with a study showing higher fatty acid content in summer and spring than in winter [112]. On the other hand, other research shows similarities with a dominant level of fatty acids in winter [117]. This research suggests that fatty acid composition can vary depending not only on the season but also on the origin of the species and the harvesting area [107,112]. Research has shown, using principal component analysis, that there is a difference with family and taxonomic rank [18,118]. Our study is the first to describe the fatty acid profile of *Halymenia durvillei* over the seasons. Considering the proportions of arachidonic acid and eicosapentaenoic acid, the period from September to December seems to be the most suitable for using this alga, depending on its fatty acid profile.

These studies suggest that fatty acid composition may vary not only by species but also by harvest area [112,117], although it should be noted that other factors may contribute to differences in results, including sampling type, sample preparation, and extraction method [119,120].

The study of seasonal variation in fatty acid composition and profile is essential to determine the best harvesting period. This study is a crucial step in highlighting the value of this species of seaweed on the Comorian coast. This study focuses on the biochemical composition of the alga *Halymenia durvillei* from the island of Comoros, but the results obtained may offer useful information for other marine algae, particularly in other tropical coastal regions.

## 4. Materials and Methods

### 4.1. Sampling

The species *Halymenia durvillei* was collected on the island of Ngazidja at Mitsamiouli in the northern region between September 2022 and August 2023. The site was located between the dragon’s back of Ivoini and the turtle island of Ndroude (latitude 11°24′22.34″ S. longitude 43°25′5.98″ E).

### 4.2. Drying and Cryo-Grinding

The harvested algae were first dried in a dark place at room temperature, and then placed in bags before being transported to the laboratory of the Institut des Substances et Organismes de la Mer. Samples were ground in liquid nitrogen.

### 4.3. Total Ashes

Total ashes were determined by a standard gravimetric method [121]. A quantity of 2 g of seaweed dry matter was incinerated in an oven at 500 °C for 8 h. The ash content was expressed as a percentage of dry weight.

### 4.4. Total Proteins

The total protein content was determined using the Kjedahl method [122]. The protein content was thus determined by the relationship between the total nitrogen content and the conversion coefficient 6.25.

The protein content is determined by the following relationship:

%P=%N×6.25

The total nitrogen content (%N) is determined according to the formula:

$$\%N=\frac{\left(V\times 14\times 100\times 0.001\times n\right)}{me}$$

V: Volume of acid solution used for titration in mL, n: normality of the sulfuric acid used for the assay, me: Mass of test sample, %N: Total nitrogen content, 14: Molar mass of nitrogen, 6.25: Nitrogen to protein conversion factor used

### 4.5. Extraction of Soluble Compounds

One gram of cryo-ground algae was suspended in 20 mL of sodium phosphate buffer (pH = 7.1, 20 mM). Extraction was performed in the dark for 20 min at 4 °C. The resulting suspension was centrifuged at 25,000× *g* for 20 min at 4 °C. The supernatant containing the water-soluble compounds was collected. The supernatant was used for the water-soluble protein assay, RPE determination, and water-soluble carbohydrate assay [39].

#### 4.5.1. Water-Soluble Proteins

Water-soluble protein content was determined according to the method of Smith et al., [123]. A quantity of 200 µL of bincinchonic acid (BCA) reagent was added to 25 µL of sample in a flat-bottomed 96-well microplate. The microplate was homogenized and incubated for 45 min at room temperature in the dark before absorbance measurement at 570 nm on a microplate reader. BSA was used as standard (range from 0 to 100 mg/L).

#### 4.5.2. Water-Soluble Carbohydrates

Water-soluble carbohydrates were determined according the modified colorimetric phenol-sulfuric acid method of Dubois et al. [124]. Phenol at 5% (200 μL) was added to 200 μL of water-soluble extract followed by 1 mL of concentrated sulfuric acid (96%). The suspension was left for 10 min at room temperature before vortexing (5 s at 3000 rpm), and then left for 15 min at room temperature and 30 min at 35 °C. Absorbance was measured at 490 nm. Glucose was used as a standard (range from 0 to 100 mg/L).

### 4.6. Polysaccharides

Extraction was carried out according to a simplified protocol adapted to different species of red algae [22]. A quantity of 1.2 g of algae powder was suspended in 40 mL of distilled water. The mixture was placed in a water bath at 90 °C with stirring for one hour. The extract obtained was centrifuged at 25,000× *g* (20 °C) for ten minutes, and the supernatant was collected. Absolute ethanol was then added to the supernatant (1:2, *v*:*v*) and vortexed. The polysaccharides precipitated were recovered by centrifugation (11500× *g*) for ten minutes at 20 °C and were placed in an oven at 60 °C for one day. The polysaccharide yield was determined by the gravimetric method.

### 4.7. Lipids and Fatty Acids

#### 4.7.1. Total Lipids

Total lipids were extracted using the modified method of Bligh and Dyer [125]. Total lipids were extracted from 2 g of dried seaweed using a mixture of dichloromethane:methanol (1:1, *v*/*v*) at room temperature for 12 h. After filtration, the maceration filtrate was recovered in a separating funnel with the addition of distilled water. The organic phase was recovered in an Erlenmeyer flask and dried by anhydrous Na_2_SO_4_. Lipid extract was obtained after removing solvents using a rotative evaporator. The lipid content was determined by gravimetric method.

#### 4.7.2. Fatty Acids (FAs)

A transesterification reaction was carried out on 10 mg of lipids with 500 µL of methanolic HCl, 300 µL of methanol, and 100 µL of chloroform in a dry bath, refluxing at 80 °C for 5 h to obtain the Fatty Acid Methyl Esters (FAMEs) in the supernatant. N-acyl pyrrolidides were prepared by direct treatment of FAMEs with pyrrolidine/acetic acid (5:1 *v*/*v*) for 60 min at 85 °C under reflux. FAME and NAP were analyzed using a GC-MS instrument (Hewlett Packard HP 6890—GC system/HP 6890—70 eV, Agilent Technologies, Santa Clara, CA, USA) equipped with an SLB-5TM column (60 m × 0.25 mm; 0.25 μm). The injector and detector temperatures were 250 °C and 280 °C, respectively. The carrier gas was helium at a flow rate of 1 mL/min. One microliter was injected in splitless mode. The column temperature was maintained at 170 °C for 4 min and increased to 300 °C at 3 °C/min for FAME analyses, and at 200 °C for 4 min and increased by 3 °C/min up to 310 °C for NAP analyses.

#### 4.7.3. Nutritional Quality Indexes

The nutritional quality indexes were calculated with the following equations:

n-6/n-3 ratio:

$$\frac{n-6}{n-3}=\frac{\sum PUFAn-6}{\sum PUFAn-3}$$

C18/C20 PUFA ratio:

$$\frac{C18}{C20}=\frac{\sum C18PUFA}{\sum C20PUFA}$$

PUFAs/SFAs:

$$\frac{PUFA}{SFA}=\frac{\sum PUFA}{\sum PUFA}$$

Atherogenicity index (AI):

$$AI=\frac{C12:0+4\times C14:0+C16:0}{\sum MUFA+\sum PUFAn-6+\sum PUFAn-3}$$

Thrombogenicity Index (TI):

$$TI=\frac{C14:0+C16:0+C18:0}{0.5\times \sum MUFA+0.5\times \sum PUFAn-6+3\times \sum PUFAn+\frac{\sum PUFAn-3}{\sum PUFAn-6}}$$

Hypocholesterolemic /Hypercholesterolemic (h/H) Fatty Acids:

$$\frac{h}{H}=\frac{C18:1n-9+C18:2n-6+C20:4n-6+C18:3n-3+C20:5n-3}{C14:0+C16:0}$$

Health-Promoting Index (HPI):

$$HPI=\frac{\Sigma UFA}{C12:0+\left(4\times C14:0\right)+C16:0}$$

Unsaturation Index (UI):

**UI =** 1** × (% monoenoics) +** 2 **× (% dienoics) +** 3 **× (% trienoics)** + **4 × (% tetraenoics) +** 5 **× (% pentaenoics) +** 6 **× (% hexaenoics)**

### 4.8. Pigments

#### 4.8.1. Determination of R-Phycoerythrin

Absorbance measurements for water-soluble extracts were carried out using the Varioskan LUX 3020-82392 reader (Thermo Fisher Scientific, Waltham, MA, USA). A quantity of 200 µL of sample was placed in a 96-well plate in triplicate. The absorbance spectrum was measured between 350 and 800 nm with an interval of 1 nm. The blank was prepared using 20mM phosphate buffer pH 7. R-Phycoerythrin (R-PE) concentrations were determined using the Sampath-Wiley (2007) [126] equation for an optical path of 1 cm.

[R − PE] = 0.1247 [(A_564_ − A_730_) − 0.4583 (A_618_ − A_730_)]

#### 4.8.2. Lipophilic Pigment Analysis

Lipophilic pigments were extracted using 1 g of cryo-ground algae suspended in a 5 mL mixture of methanol/ammonium acetate (20 g/L). The suspension was vortexed for 30 s, stored at −20 °C for 15 min, and centrifuged at 2000× *g* for 5 min at 4 °C. The supernatant was filtered through a regenerated cellulose syringe filter (0.45 μm). Carotenoids and chlorophylls were quantified by HPLC. The chromatographic equipment was a WATERS e2695 apparatus (Milford, MA, USA) comprising a pump system, an automatic injector, and a detection system consisting of a diode array spectrophotometer (WATERS 2998 PDA). The pigments were separated on a Waters SunFire C18 3.5 4.6 × 100 mm analytical column (1 mL/min flow rate) using a mobile phase composed of 2 solvents. Solvent A was methanol and ammonium acetate (1 mg/L) (80/20, *v*/*v*) and solvent B was methanol and acetone (60/40, *v*/*v*). The elution gradient consisted of 3 stages: 20% of B to 100% of B for 10 min, then isocratic 100% of B for 10 min, and return to initial conditions for 10 min. Chlorophyll pigments and carotenoids were detected at a wavelength of 440 nm and characterized by their retention time and spectrum between 400 and 800 nm. The quantification was realized by external calibration.

### 4.9. Environmental Parameters

Environmental parameters, precipitation, and temperature from September 2022 to August 2023 were obtained via access to Copernicus satellite data (https://data.marine.copernicus.eu/products (accessed on 1 March 2024)).

### 4.10. Statistical Analysis

Statistical analyses were performed using past04 software (version 4.12). Data normality (Shapiro) and homogeneity of variance were tested. ANOVA (analysis of variance) and HSD Tukey’s test were performed if normality was confirmed. If not, non-parametric tests were used (Kruskal–Wallis and Dunn’s tests). All analyses were performed in triplicate.

R.4.1.0/RStudio software was used to perform principal component analysis on the fatty acids and grouping, allowing the differences between the groups to be clearly identified.

## 5. Conclusions

This study shows for the first time the biochemical composition of Comorian seaweed, *Halymenia durvillei*, with a pattern of change depending on the harvesting period. *Halymenia durvillei* is rich in polysaccharides, which can be used as a source of energy for human well-being. The presence of the eicosapentaenoic and arachidonic essential fatty acids also makes these algae a good source of health and nutrition. The warm wet season and the start of the dry season appear to be the most profitable times to harvest seaweed, due to its high polysaccharide and protein content. While this study focuses primarily on the lipid profile of these red algae, it is indeed crucial to examine the amino acid, carbohydrate, and mineral compositions in future studies. These elements will provide a better understanding of the nutritional potential of this alga. However, future prospects could include a detailed study of biological activities to improve our knowledge of this alga species in Comoros. Another part of future trends should be focused on the societal acceptance of the seaweed economy.

## Figures and Tables

**Table 1:** Biochemical composition of *Halymenia durvillei*. The data presented are the mean and standard deviation of 36 determinations, except for pigments (mean of 30 determinations).

Compound	Content
Ashes ^a^	35.59 ± 2.55
Total proteins ^a^	13.93 ± 0.09
Water-soluble proteins ^a^	0.74 ± 0.20
Water-soluble carbohydrates ^a^	1.52 ± 0.69
Polysaccharides ^a^	35.09 ± 6.14
Lipids ^a^	0.27 ± 0.02
Chlorophyll ^ab^	1.49 ± 1.29
Carotenoids ^b^	0.09 ± 0.07
R-phycoerythrin ^b^	140 ± 103

a = %dw; b = µg/g dw.

**Table 2:** Fatty acid composition (% of total fatty acids—data presented are the mean of 36 determinations and standard deviation).

Fatty Acids	% of Total Fatty Acids (TFAs)
C14:0 (Myristic)	1.97 ± 0.38
C15:0 (Pentadecanoic)	2.0 ± 0.36
C16:0 (Palmitic)	43.33 ± 6.72
C17:0 (Heptadecanoic)	1.35 ± 0.57
C18:0 (Stearic)	5.9 ± 1.84
C19:0 (Nonadecanoic)	<0.1
C20:0 (Arachidic)	<0.2
C21:0 (Heneicosanoic)	<0.1
C22:0 (Behenic)	<0.3
C23:0 (Tricosanoic)	<0.2
C24:0 (Lignoceric)	<0.6
**Total SFAs**	**55.1 ± 5.2**
C16:1 (Palmitoleic)	9.05 ± 2.7
C17:1 (Margaroleic)	2.39 ± 1.51
C18:1n-9 (Oleic)	11.58 ± 2.73
C19:1 (Nonadecenoic)	<0.5
C20:1 (Eicosenoic)	<0.2
C22:1 (Erucic)	<0.3
**Total MUFAs**	**23.4 ± 2.4**
C18:2n-6 (Linoleic)	2.39 ± 2.13
C20:3 (Eicostrienoic)	<0.2
C20:4n-6 (Arachidonic)	1.87 ± 1.4
C20:5n-3 (Eicosapentaenoic)	1.46 ± 1.36
**Total PUFAs**	**5.8 ± 3.0**
**U.I FA**	**15.7 ± 3.8**

SFA: Saturated fatty acids; MUFA: Monounsaturated fatty acids; PUFA: Polyunsaturated fatty acids; U.I FA: Unidentified fatty acids.

**Table 3:** Nutritional quality indexes of fatty acids from *Halymenia durvillei.*

Seaweed	n-6/n-3	C18/C20 PUFA	PUFAs/SFAs	AI	TI	h/H	HPI	UI
*H. durvillei*	2.84	0.69	0.1	1.76	2.74	0.38	0.57	43.32

**Table 4:** Seasonal monitoring of fatty acids *Halymenia durvillei* (n = 3) (% of total fatty acids). Significant differences at *p* < 0.05 are indicated by different letters.

FA %	2022				2023							
	September	October	November	December	January	February	March	April	May	June	July	August
C14:0	1.57 ± 1.37 ^a^	1.78 ± 0.14 ^a^	2.12 ± 0.17 ^b^	2.59 ± 0.22 ^c^	2.2 ± 0.19 ^b^	1.93 ± 0.37 ^abc^	1.69 ± 0.08 ^a^	1.74 ± 0.14 ^ab^	2.16 ± 0.07 ^ab^	2.2 ± 0.08 ^ab^	2.4 ± 0.27 ^abc^	1.25 ± 0.08 ^a^
C15:0	1.53 ± 0.48 ^a^	1.78 ± 0.11 ^a^	1.56 ± 0.04 ^a^	1.91 ± 0.1 ^a^	2.42 ± 0.21 ^b^	2.68 ± 0.18 ^b^	2.31 ± 0.07 ^b^	2.35 ± 0.07 ^b^	1.94 ± 0.19 ^a^	1.97 ± 0.17 ^a^	1.93 ± 0.15 ^a^	1.66 ± 0.08 ^a^
C16:1	5.43 ± 0.62 ^a^	6.89 ± 0.9 ^b^	5.33 ± 0.14 ^a^	7.59 ± 0.47 ^b^	8.43 ± 0.98 ^c^	10.06 ± 0.7 ^d^	13.46 ± 0.29 ^e^	13.76 ± 0.61 ^e^	9.3 ± 1.93 ^cd^	8.25 ± 0.94 ^bc^	10.51 ± 0.61 ^d^	9.62 ± 0.24 ^c^
C16:0	45.22 ± 1.77 ^a^	44.78 ± 1.22 ^a^	49.83 ± 3.2 ^b^	41.71 ± 1.75 ^c^	29.97 ± 0.26 ^d^	34.67 ± 2.12 ^e^	41.92 ± 1.09 ^c^	40.97 ± 1.24 ^c^	45.3 ± 1.22 ^a^	43.49 ± 1.83 ^c^	45.49 ± 2.09 ^ac^	56.63 ± 0.41 ^f^
17:1	1.28 ± 0.28 ^a^	2.44 ± 1.26 ^b^	0.2 ± 0.19 ^c^	0.46 ± 0.03 ^c^	5.09 ± 0.66 ^d^	4.89 ± 0.3 ^d^	2.92 ± 0.47 ^e^	3.23 ± 0.29 ^e^	1.67 ± 0.11 ^a^	1.99 ± 0.19 ^ab^	2.26 ± 0.09 ^b^	2.3 ± 0.1 ^b^
C17:0	0.66 ± 0.05 ^a^	0.68 ± 0.04 ^a^	0.64 ± 0.02 ^a^	1.57 ± 0.15 ^b^	2.19 ± 0.23 ^c^	2.2 ± 0.09 ^c^	1.37 ± 0.08 ^b^	1.75 ± 0.36 ^bc^	1.72 ± 0.3 ^bc^	1.33 ± 0.21 ^b^	1.33 ± 0.03 ^b^	0.76 ± 0.02 ^a^
C18:2n6	1.4 ± 0.57 ^a^	0.77 ± 0.2 ^b^	0.81 ± 0.07 ^b^	1.22 ± 0.45 ^ab^	5.54 ± 0.77 ^c^	2.73 ± 0.7 ^d^	1.84 ± 0.27 ^a d^	1.79 ± 0.1 ^ad^	4.89 ± 0.67 ^c^	6.69 ± 1.27 ^c^	0.86 ± 0.11 ^ab^	0.18 ± 0.02 ^e^
C18:1n9	15.72 ± 0.73 ^a^	14.32 ± 1.86 ^a^	17.19 ± 0.25 ^b^	12.19 ± 0.61 ^c^	9.13 ± 1.36 ^d^	9.73 ± 0.82 ^d^	10.69 ± 0.64 ^d^	10.27 ± 0.22 ^d^	10.2 ± 0.68 ^d^	11.14 ± 0.76 ^c d^	9.24 ± 0.42 ^d^	9.17 ± 0.16 ^d^
C18:0	5.89 ± 0.23 ^a^	4.7 ± 0.24 ^b^	5.21 ± 0.33 ^b^	7.86 ± 0.48 ^c^	8.79 ± 1.22 ^d^	5.2 ± 0.69 ^ab^	3.99 ± 0.27 ^b^	4.06 ± 0.2 ^b^	8.2 ± 2.49 ^cd^	6.69 ± 0.7 ^ac^	7.17 ± 0.66 ^cd^	3.04 ± 0.03 ^f^
C19:1	-	-	-	-	1.47 ± 0.38 ^a^	0.64 ± 0.55 ^b^	-	-	-	-	-	-
C19:0	-	-	-	-	0.13 ± 0.11	-	-	-	-	-	-	-
C20:3	-	-	-	-	-	-	0.15 ± 0.01 ^a^	0.18 ± 0.01 ^a^	0.06 ± 0.02 ^b^	0.07 ± 0.01 ^b^	0.1 ± 0.04 ^c^	0.01 ± 0.01 ^d^
C20:4n-6	2.35 ± 0.54 ^a^	2.64 ± 0.46 ^a^	3.01 ± 0.37 ^b^	5.5 ± 0.05 ^c^	1 ± 0.06 ^d^	1.28 ± 0.19 ^d^	1.49 ± 0.09 ^d^	1.45 ± 0.29 ^d^	1.55 ± 0.1 ^d^	1.45 ± 0.43 ^d^	0.55 ± 0.24 ^e^	0.21 ± 0.03 ^f^
C20:5n-3	1.7 ± 0.36 ^a^	2.26 ± 0.35 ^b^	2.48 ± 0.33 ^b^	5.16 ± 0.14 ^c^	0.46 ± 0.02 ^d^	0.56 ± 0.1 ^d^	0.77 ± 0.03 ^de^	0.97 ± 0.1 ^e^	1.13 ± 0.3 ^ae^	1.15 ± 0.12 ^ae^	0.63 ± 0.12 ^de^	0.2 ± 0.04 ^f^
C20:1	0.13 ± 0.22 ^a^	-	0.41 ± 0.25 ^b^	0.06 ± 0.11 ^c^	0.37 ± 0.24 ^b^	0.08 ± 0.14 ^c^	-	-	-	-	-	-
C20:0	-	-	0.05 ± 0.08 ^a^	0.26 ± 0.01 ^b^	0.38 ± 0.08 ^c^	0.17 ± 0.15 ^d^	-	-	-	-	-	-
C21:0	-	-	-	-	0.07 ± 0.06	-	-	-	-	-	-	-
C22:1	0.1 ± 0.17 ^a^	-	0.47 ± 0.32 ^b^	0.13 ± 0.23 ^c^	0.49 ± 0.09 ^b^	0.14 ± 0.24 ^c^	-	-	-	-	-	-
C22:0.	-	-	-	-	-	-	0.06 ± 0.06 ^a^	0.12 ± 0.02 ^b^	0.57 ± 0.08 ^c^	0.44 ± 0.16 ^d^	0.4 ± 0.05 ^d^	0.03 ± 0.01 ^a^
C23:0	-	-	-	0.48 ± 0.05 ^a^	0.31 ± 0.08 ^b^	0.15 ± 0.13 ^c^	-	-	-	-	-	-
C24:0	0.14 ± 0.12 ^a^	0.2 ± 0.07 ^ab^	0.29 ± 0.01 ^b^	1.02 ± 0.1 ^d^	1 ± 0.11 ^d^	0.75 ± 0.12 ^e^	-	-	-	-	-	-
C26:0	0.03 ± 0.05	-	-	-	-	-	-	-	-	-	-	-
SFAs	55.04 ± 4.0 ^a^	53.92 ± 1.2 ^a^	59.7 ± 3.85 ^b^	57.4 ± 2.85 ^ab^	47.46 ± 2.5 ^c^	47.75 ± 3.85 ^c^	51.34 ± 1.6 ^d bc^	50.99 ± 2.0 ^bc^	59.89 ± 4.3 ^b^	56.12 ± 3.16 ^ab^	55.14 ± 3.2 ^b^	63.37 ± 0.6 ^b^
MUFAs	22.66 ± 2.0 ^a^	23.65 ± 1.02 ^a^	23.6 ± 1.15 ^a^	20.43 ± 1.4 ^b^	24.98 ± 3.71 ^a^	25.54 ± 2.75 ^ac^	27.07 ± 1.41 ^c^	27.26 ± 1.13 ^c^	21.17 ± 2.72 ^ab^	21.38 ± 1.89 ^ab^	23.4 ± 1.12 ^ab^	21.09 ± 0.5 ^ab^
PUFAs	5.45 ± 1.46 ^a^	5.67 ± 0.56 ^a^	6.3 ± 0.78 ^b^	11.88 ± 0.65 ^c^	7 ± 0.85 ^b^	4.57 ± 0.99 ^ab^	4.25 ± 0.39 ^ab^	4.39 ± 0.5 ^ab^	7.63 ± 1.09 ^bc^	9.36 ± 1.83 ^bc^	2.14 ± 0.51 ^d^	0.6 ± 0.1 ^e^
Σ n-6	3.75 ± 1.11 ^a^	3.41 ± 0.39 ^a^	3.82 ± 0.44 ^a^	6.72 ± 0.5 ^b^	6.54 ± 0.83 ^b^	4.01 ± 0.89 ^a^	3.33 ± 0.36 ^a^	3.24 ± 0.39 ^a^	6.44 ± 0.77 ^b^	8.14 ± 1.7 ^c^	1.41 ± 0.35 ^ab^	0.39 ± 0.05 ^d^
Σ n-3	1.7 ± 0.36 ^a^	2.26 ± 0.17 ^b^	2.48 ± 0.33 ^b^	5.16 ± 0.14 ^c^	0.46 ± 0.02 ^d^	0.56 ± 0.1 ^d^	0.92 ± 0.04 ^de^	1.15 ± 0.11 ^ae^	1.19 ± 0.32 ^e^	1.22 ± 0.13 ^ae^	0.73 ± 0.15 ^d^	0.21 ± 0.05 ^f^

**Table 5:** Seasonal monitoring of nutritional indexes.

Indexes	2022				2023							
	September	October	November	December	January	February	March	April	May	June	July	August
n-6/n-3	2.07	1.5	1.54	1.3	14.14	7.3	3.61	2.85	5.82	6.75	1.93	1.66
C18/C20 PUFA	0.37	0.16	0.15	0.11	3.14	1.4	0.81	0.75	1.85	2.56	0.75	0.6
PUFAs/SFAs	0.1	0.11	0.11	0.21	0.15	0.1	0.08	0.09	0.13	0.17	0.04	0.01
AI	1.84	1.77	1.99	1.62	1.25	1.42	1.55	1.52	1.88	1.71	2.29	2.85
TI	2.83	2.45	2.67	1.75	2.44	2.54	2.61	2.46	3.21	2.83	3.83	5.06
h/H	0.45	0.43	0.45	0.55	0.5	0.39	0.34	0.34	0.37	0.45	0.24	0.17
HPI	0.55	0.51	0.51	0.62	0.81	0.71	0.64	0.66	0.53	0.59	0.44	0.35
UI	43.34	42.86	49.68	70.71	42.39	38.94	41.04	42.02	42.98	46.54	29.39	23.03

## Data Availability

Data are contained within the article.
